# *In vivo* pH monitoring using boron doped diamond microelectrode and silver needles: Application to stomach disorder diagnosis

**DOI:** 10.1038/srep03257

**Published:** 2013-11-19

**Authors:** Stéphane Fierro, Ryo Seishima, Osamu Nagano, Hideyuki Saya, Yasuaki Einaga

**Affiliations:** 1 Department of Chemistry, Faculty of Science and Technology, Keio University, 3-14-1 Hiyoshi, Yokohama 223-8522, Japan; 2 Division of Gene Regulation, Institute for Advanced Medical Research, Keio University, 35 Shinanomachi Shinjuku, Tokyo 160-8582, Japan; 3 JST, CREST, 5, Sanbancho, Chiyoda-ku, Tokyo 102-0075, Japan

## Abstract

This study presents the *in vivo* electrochemical monitoring of pH using boron doped diamond (BDD) microelectrode and silver needles for potential application in medical diagnosis. Accurate calibration curve for pH determination were obtained through *in vitro* electrochemical measurements. The increase induced in stomach pH by treatment with pantoprazole was used to demonstrate that it is possible to monitor the pH *in vivo* using the simple and noninvasive system proposed herein. Using the results of the *in vivo* and *in vitro* experiments, a quantitative analysis of the increase in stomach pH is also presented. It is proposed that the catheter-free pH monitoring system presented in this study could be potentially employed in any biological environment.

Changes in pH affect various physiological and pathological conditions within our body. In particular, dysregulated pH in human tumors was shown to be involved in tumor progression and malignancy^1^. Cancer cells often manifest the expression of several types of acid extruders including Na^+^ - H^+^ exchanger-1 (NHE1), H^+^-ATPases and monocarboxylate transporter (MCTs) and thereby create an acidic tumor microenvironment^2^. Acidification of tumor microenvironment increases cellular motility and enhances the activities of various proteases which are responsible for the degradation of extracellular matrix and leads to cancer cells invasion and metastasis^1^,^3^. Furthermore, cellular pH gradient in tumor due to the low extracellular pH and higher intracellular pH was shown to influence the electrical charge of drug and impair the transport and action of anticancer drugs^4^. Recently, proton pump inhibitors (PPIs) were shown to normalize the dysregulated pH in tumor microenvironment and, therefore, sensitize cancer cells to anticancer drugs^5^,^6^. These findings suggest that the use of noninvasive *in vivo* pH monitoring within tumor tissue provides essential information for improving the efficacy of combination chemotherapy with anticancer drugs and PPI.

Typically, pH monitoring is made through the use of indicator reagents, pH test strips, metal electrodes (hydrogen, quinhydrone or antimony electrodes) or glass electrodes^7^. The latter method currently is the most commonly used electrode for pH measurements because of the high selectivity, reliability and pH range available^7^,^8^,^9^,^10^. Glass electrodes, however, are not well suited for miniaturization^11^ and the fragile nature of glass can be problematic for *in vivo* measurements. In the recent years, several improvements have been made towards the *in vivo* monitoring of pH inside the stomach. The first novel technique involved a catheter, which consists of inserting a pH sensitive electrode mounted on a plastic tube through the subject's mouth and down into the stomach^12^. This procedure can be quite invasive for the patient. Nowadays, the gold standard in stomach pH monitoring is the Bravo® pH monitoring system, which is a capsule-based, patient-friendly system where a miniature pH capsule attached to the esophagus wirelessly transmits pH data to a small recorder worn on a shoulder strap or waistband^13^,^14^. The capability of the Bravo® system to measure pH in any biological environment, however, remains unclear. Therefore, the development of alternative noninvasive methods for *in vivo* pH monitoring is warranted.

BDD electrodes have attracted much attention in the recent years owing to their superior properties compared to other conventional electrode materials (i.e. glassy carbon or platinum electrodes). In addition to the well-known properties of diamond, such as high thermal conductivity and extreme hardness, the attractive features of BDD include a wide potential window for water stability, low background capacitive current and chemical inertness strong resistance against adsorption^15^,^16^. The latter property makes BDD electrodes an appropriate candidate for pH monitoring. A recent study further showed that accurate calibration curves could be obtained using BDD electrodes and chronopotentiometry measurements^17^. In their study, however, the application of BDD electrodes for *in vivo* pH monitoring was not evaluated.

In this work, the *in vivo* monitoring of pH through the use of BDD microelectrodes conjointly with chronopotentiometry measurements is presented. Additionally, thin silver needles (i.e., acupuncture needles) were used as counter and reference electrodes. The capabilities of a silver needle treated anodically in HCl as reference electrode for *in vivo* experiments is also illustrated. The effects of pantoprazole, a proton pump inhibitor, on the stomach pH are presented to demonstrate that owing to the exceptional characteristics of BDD, the system proposed herein could be adapted for *in vivo* pH measurements.

## Results

### *In vitro* experiments

**Figure 1:**
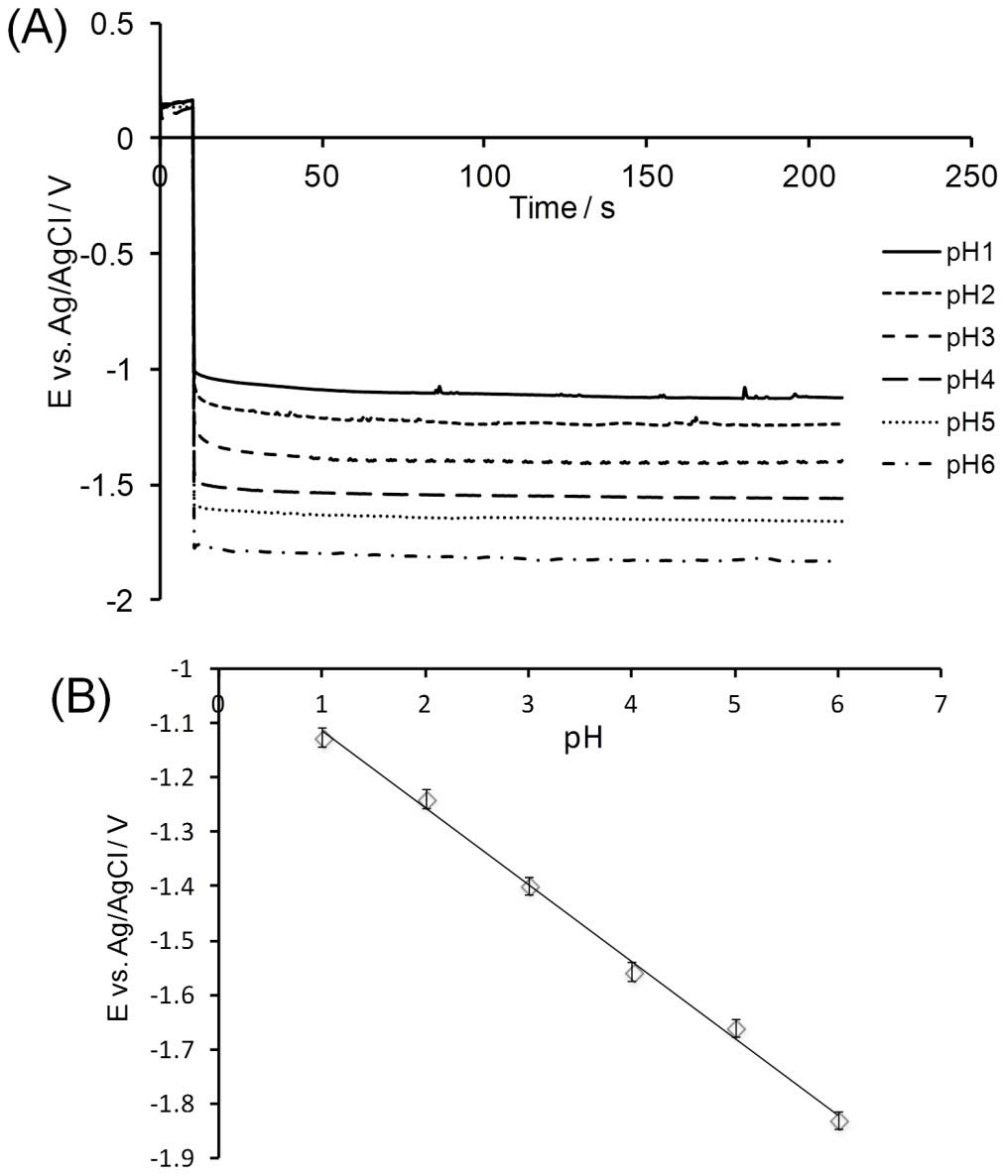
*In vitro* pH calibration. (A) Chronopotentiometry measurements recorded in FIXANAL® buffer (from pH 1 to pH 6) for a current step of −50 nA. The working electrode was a BDD microelectrode (0.1% B/C), the counter electrode was an acupuncture silver needle previously treated anodically for one minute in HCl (1 M) at 2 V vs Ag/AgCl sat. and the counter electrode was another silver acupuncture needle (untreated). 0 A was applied for 5 seconds in order to stabilize the potential before applying −50 nA. (B) Calibration curve for pH detection: the potential reported from (A) was plotted versus pH. T = 37°C.

Figure 1A displays chronopotentiometry measurements recorded on BDD microelectrode in FIXANAL® buffers from pH 1 to pH 6. For these experiments, 0 A was applied for 10 seconds before performing the desired current step (from 0 to −50 nA in Figure 1A). Although the reference electrode is a silver acupuncture needle treated anodically in HCl, Figure 1A shows that the open circuit potential (recorded at 0 A) is similar with respect to commercially available Ag/AgCl sat. reference electrodes (between 0.15 and 0.2 V). It is also relevant to mention that these experiments were performed at 37°C in order to simulate *in vivo* conditions. Figure 1A shows that the potential recorded following a 50 nA negative current step decreases with increasing values of pH. In fact, the negative current step induces the production of hydrogen gas from water at the surface of the electrode via the following reaction: 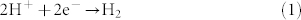
Using this feature, a calibration curve for pH determination can be constructed. An example of such curve for a given current step of −50 nA is presented in Figure 1B. The latter figure shows that an accurate pH calibration curve (slope: −1.41·10^−1^ ± 0.04·10^−1^, r^2^: 0.996) could be obtained for the current step investigated. As discussed earlier in^17^, the known chemical inertness of BDD is the chief reason for the exceptional accuracy of the linear relationship of the pH calibration curves relative to other electrode materials.

The slopes of the pH calibration curves are in good agreement with the results reported using BDD macroelectrode in^17^.

As reported in^17^, however, the primary limitation of this method is the presence of electrochemically active compounds, which could induce unwanted electrochemical reactions at the surface of the electrode. Such compounds are likely to be found in a living organ. To avoid this undesirable effect^17^, recommended to apply a higher current step to deplete the active compound in the vicinity of the working electrode such that the potential recorded mainly refers to the hydrogen evolution reaction.

If the applied current is too high in absolute value, the high H_2_ production rate could induce a local artificial pH change in the vicinity of the electrode surface, which could significantly alter the accuracy of the pH measurements.

Due to the aforementioned reasons and based on previously reported results^17^, the current step selected for all the *in vivo* experiments presented in this work was set to −50 nA.

### *In vivo* experiments

**Figure 2:**
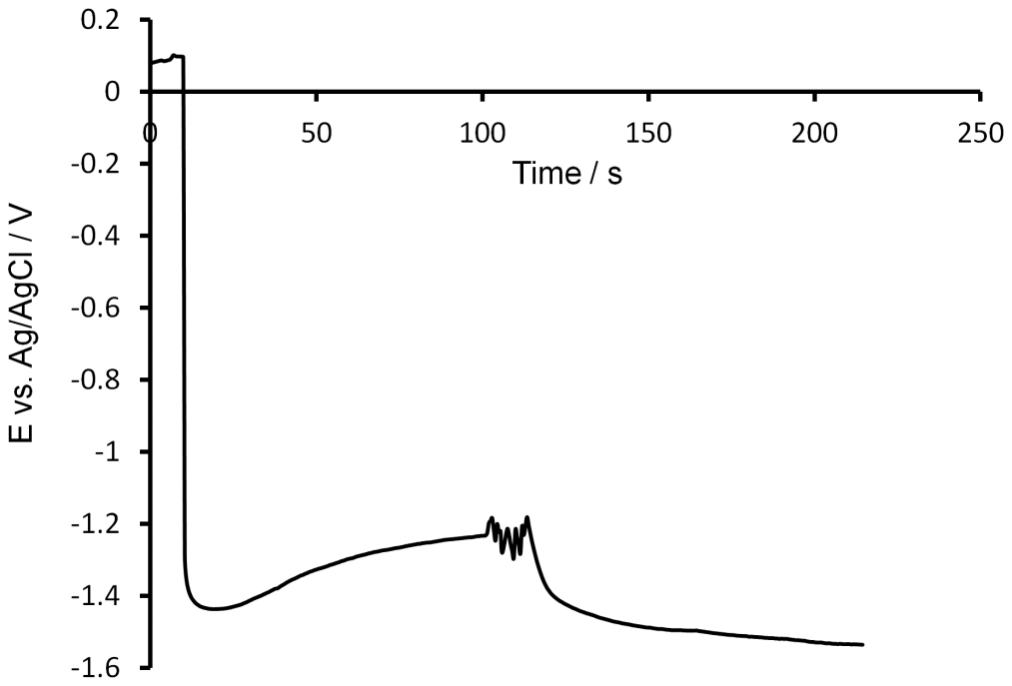
Evaluation of the response of the BDD pH sensor inside the stomach of a healthy mouse. Chronopotentiometry measurements: The open circuit potential (0 A) was recorded for 10 seconds before applying a current step of −50 nA for 215 seconds. The working electrode was a BDD microelectrode (0.1% B/C), the counter electrode was an acupuncture silver needle previously treated anodically for one minute in HCl (1 M) at 2 V vs Ag/AgCl sat. and the counter electrode was another silver acupuncture needle (untreated). At approximately 100 seconds during the experiment, a small volume of 0.1 M PBS (pH = 7.45) was introduced inside the stomach using a syringe. T = 37°C.

Although the *in vitro* measurements presented in Figure 1 appear promising for the potential use of this technique for *in vivo* pH measurements, it was necessary to determine whether or not a pH change occurring in a living organ (such as the stomach) could be detected. Towards this goal, a chronopotentiometry measurement was performed in the stomach of a healthy mouse. During the recording of the potential at a 50 nA current step, a small volume of 0.1 M PBS solution was injected inside the stomach using a syringe. Figure 2 shows the result of this experiment and the change in potential following the injection of PBS can clearly be noticed. Prior the injection at 100 seconds, the potential measured seems to stabilize toward a value, which, based on the calibration shown in Figure 1 corresponds to an acidic pH. Shortly after injection, however, the trend changes and the potential start decreasing towards values corresponding to more alkaline pH. Therefore, this measurement demonstrates that the BDD electrode is also sensitive to pH changes within a living organ. It is relevant to point out that the potential recorded at i = 0 A is about 0.15 V, which indicates/suggests that the silver acupuncture needle anodically treated in HCl works efficiently as Ag/AgCl reference electrode even under *in vivo* conditions. The AgCl layer at the surface of the needle might also be regenerated by the Cl^−^ anions present within the living tissues. For these reasons, the use of silver acupuncture needles as reference and counter electrodes is appropriate for *in vivo* electrochemical experiments in confined spaces. This capability is important, given that accurate chronopotentiometry measurements are difficult to obtain with an unreliable reference electrode.

Gastric acid secretion by parietal cells inside the stomach wall is regulated by an enzyme (H^+^/K^+^ -ATPase), which pumps protons in exchange of potassium ions that pass through the apical membrane^18^,^19^. Pantoprazole is a potent H^+^/K^+^ -ATPase inhibitor and thus inhibits gastric acid secretion, which induces a pH increase in the stomach^18^,^19^.

To evaluate the effect of pantoprazole on stomach pH, five mice were treated with 40 mg/kg for a five-day period. The pH in the stomach was then determined via chronopotentiometry measurements.

For the sake of comparison and completeness, the pH in the stomach of five non-treated control mice was also determined and each chronopotentiometry measurement was recorded twice in a row to attest the reproducibility of the results.

**Figure 3:**
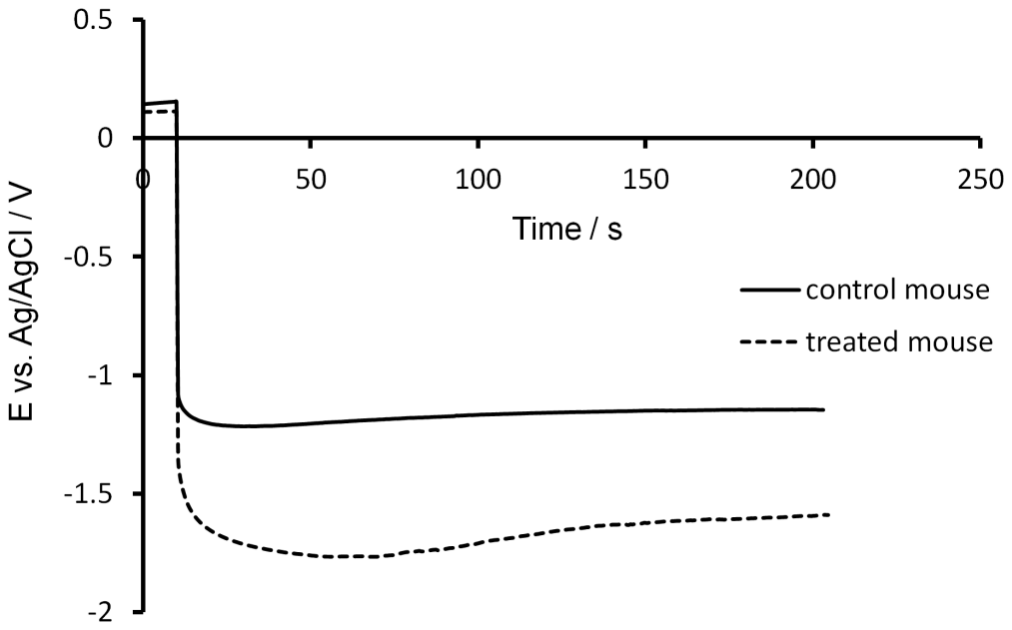
Evaluation of the effects of pantoprazole on the stomach pH: Difference between a healthy mouse and another specimen treated for 5 days. Chronopotentiometry measurements: The open circuit potential (0 A) was recorded for 10 seconds before applying a current step of −50 nA for 200 seconds. The working electrode was a BDD microelectrode (0.1% B/C), the counter electrode was an acupuncture silver needle previously treated anodically for one minute in HCl (1 M) at 2 V vs Ag/AgCl sat. and the counter electrode was another silver acupuncture needle (untreated). T = 37°C.

Figure 3 illustrates a typical difference in potential measured between the control and pantoprazole treated mouse. The potential measured inside the mice treated with pantoprazole is higher in absolute value than the potential recorded inside the stomach of the non-treated mice. This indicates that the pantoprazole induced an increase of the stomach pH.

It is worthwhile to document that the potential recorded at i = 0 A during the first ten seconds of the experiments (about 0.15 V) remains quite similar to the standard potential of an Ag/AgCl reference electrode This proves that the use of treated silver needles as reference electrode for *in vivo* electrochemical measurements was appropriate.

To provide a quantitative estimation of the pH increase, the average potential corresponding to the control mice was computed at −1.23 ± 0.1 V vs Ag/AgCl. Based on Figure 1B, the pH is estimated at 1.8 ± 0.3, which is in reasonable agreement with the expected value inside the stomach of a healthy mouse.

This procedure was repeated to also determine the increase in stomach pH of the mice treated induced by five days treatment with pantoprazole. The results are summarized in Table 1. A significant increase in stomach pH was observed for four out of five mice treated with pantoprazole. Previous studies focusing on the effects of this drug have shown that its efficiency can vary substantially from one specimen to another^18^, which could explain the disparity of the results presented in Table 1.

However, Table 1 shows that for one specimen (mouse 4), no increase in stomach pH could be achieved. This discrepancy could for instance arise from an inaccurate measurement or to a resistance of that particular specimen against pantoprazole. This strongly suggests that the technique proposed herein to measure pH *in vivo* can be further improved, which will be deferred to future work.

## Discussion

In the present study, *in vitro* and *in vivo* pH measurements were performed through the use of BDD microelectrodes and silver acupuncture needles conjointly with chronopotentiometry measurements. Potential applications for stomach cancer diagnosis or other stomach disorders was used as a typical example to illustrate the capabilities of this new system for *in vivo* pH monitoring.

It is demonstrated herein that highly accurate calibration curves for pH can be constructed using BDD microelectrode and chronopotentiometry measurements. Moreover, the use of silver needle treated anodically in HCl as reference electrode was proven to be a very efficient system for *in vitro* and *in vivo* electrochemical measurements. Using this technique, it was possible to study *in vivo* pH variations and assess the effects of pantoprazole, a potent gastric proton pump inhibitor, by measuring directly the pH inside the stomach. This new catheter-free, patient-friendly technique could be potentially used to monitor pH in any biological environment. The possibility to couple this technique with a wireless data acquisition system can also be envisaged.

## Methods

### Chemicals and materials

FIXANAL® buffers (from pH 1 to pH 6) were purchased from Fluka analytical. Disodium hydrogen phosphate dodecahydrate, disodium hydrogen phosphate dehydrate, HCl, acetone and sulfuric acid were purchased from Wako. All *in vivo* experiments were performed by using C57BL/6J mice purchased from CLEA Japan. Pantoprazole was purchased from Sigma Aldrich Japan, used by dissolving in water. Chemicals were used without further purification.

### Preparation of BDD microelectrodes

**Figure 4:**
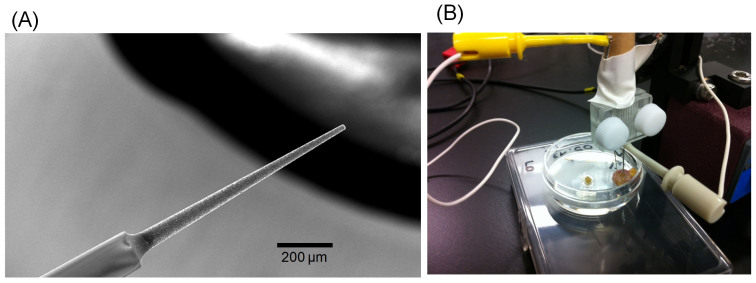
(A) SEM image of the BDD microelectrode. The image shows the tip of the needle shaped BDD microelectrode. (B) Photo image of *in vivo* experimental set up.

BDD microelectrodes were prepared using a microwave plasma-assisted chemical vapor deposition (MPCVD) set-up (ASTeX Corp.). Acetone was used as a carbon source, and B(OCH_3_)_3_ as a source of boron. The concentration of the latter was 0.1% w/w in the source. The surface morphology and crystalline structures were characterized using scanning electron microscopy (SEM). Figure 4A displays a SEM image of the BDD microelectrode. BDD was deposited on a tungsten needle (20 μm in diameter) in an MPCVD chamber at 2.5 kW using high-purity hydrogen as a carrier gas. A portion of the needle was isolated using a glass capillary in order to define the working surface area so that about between 0.5 and 1 mm remains uncovered by the capillary. The film quality was confirmed by Raman spectroscopy (not shown). The BDD microelectrodes were pre-treated by ultrasonication in 2-propanol for about 10 minutes followed by rinsing with high-purity water to remove any organic impurities that may have remained within the BDD film after deposition in the MPCVD chamber.

### Electrochemical measurements

All electrochemical measurements were carried out using an AUTOLAB PGSTAT potentiostat at 37°C. The reference electrode was a silver made acupuncture needle (50 μm in diameter), which was anodically treated in 1 M HCl (2 V vs. Ag/AgCl sat.) for 1 minute in order to form AgCl at the needle's surface. The counter electrode was another silver made acupuncture needle (untreated) and the working electrode was the BDD microelectrode. Considering the tungsten needle as a cylinder, the working geometric area was about 6.3·10^−4^ cm^2^. All potentials quoted in this work are with respect to the Ag/AgCl reference electrode (0.2 V vs. SHE).

### *In vivo* experiments

A preliminary test was performed in the stomach of a healthy mouse in order to verify if the BDD electrode was sensitive to pH changes occurring in a living organ: the potential was recorded and during the measurement, a small amount of 0.1 M PBS was injected using a syringe inside the stomach. In order to measure *in vivo* the pH, the BDD needle and the two silver acupuncture needles (HCl treated and non-treated) were inserted in the stomach to be analyzed to a depth between 2 and 3 mm (Figure 4B). Concerning the pantoprazole experiments, the drug was administered (40 mg/kg) to 5 mice for 5 days. After these 5 days, the stomach was withdrawn from the mice and the pH was measured using a BDD microelectrode. These measurements were performed also on 5 control mice in order to evaluate the effects of pantoprazole. All animal experiments were performed in accordance with protocols approved by the Ethics Committee of Keio University.

## Author Contributions

Y.E., H.S., O.N. and S.F. conceived the project and designed the experiments. R.S. and S.F. performed the experiments. S.F. analyzed the data. S.F. wrote the manuscript. All authors reviewed the manuscript.

## Figures and Tables

**Table 1:** Numeric estimation of the stomach pH difference observed between a healthy and another mouse treated with pantoprazole

mouse	1	2	3	4	5
pH (control)	1.8	1.8	1.8	1.8	1.8
pH after treated with pantoprazole	6	4.4	5.4	1.1	3.2
Increased in pH compared to control mice	4.2	2.6	3.6	-	1.4

The pH calibration curve presented in Figure 1B was used in order to convert the potential measured under the same conditions in the stomach of five control mice (untreated) and inside the stomach of five mice, which were treated for five days with pantoprazole (40 mg/kg).
